# Individualized Lifestyle Change For Every Aging Brain (InLife): Harmonization of participant‐level data from randomized controlled trials of lifestyle behaviors to impact cognitive outcomes

**DOI:** 10.1002/alz70860_098604

**Published:** 2025-12-23

**Authors:** Shannon Halloway, Michael E Schoeny, Laura D Baker, Zoe Arvanitakis, Heather Hrynk, David X. Marquez, Nathan Tintle

**Affiliations:** ^1^ University of Illinois Chicago College of Nursing, Chicago, IL, USA; ^2^ Rush University College of Nursing, Chicago, IL, USA; ^3^ Wake Forest University School of Medicine, Winston‐Salem, NC, USA; ^4^ Rush Alzheimer's Disease Center, Rush University Medical Center, Chicago, IL, USA; ^5^ Department of Kinesiology and Nutrition, University of Illinois Chicago, Chicago, IL, USA

## Abstract

**Background:**

Lifestyle health behaviors, such as physical activity (PA), cognitive activity (CA), healthy diet (HD), and social activity (SA), may help reduce risk of cognitive decline and Alzheimer's disease and related dementias (ADRD). However, supporting evidence across both observational and experimental studies is inconsistent. Participant‐level data harmonization across existing studies offers (a) improved power; (b) the opportunity to address heterogeneity in participant characteristics, health behaviors, and cognitive outcomes with precision; and (c) focus on behavior change over time vs. trial efficacy.

**Method:**

The purpose of *InLife* (Individualized Lifestyle Change **F**or **E**very Aging Brain) is to harmonize existing participant‐level data from large randomized controlled trials (RCTs) to examine how intervention‐related changes in health behavior impact cognitive outcomes across diverse groups of older adults. We will also identify the type(s) of behavior and magnitude of behavior change that have the greatest impact, and if impact varies by determinants of health.

**Result:**

To date, a total of 12 U.S. trials focused on improving cognitive function by modifying ≥1 lifestyle health behaviors (PA, CA, HD, SA) will contribute participant‐level data (*N*>9,500 older adults). We will begin by obtaining data from all trials (all participants; all time points). We will then harmonize data using a scalable item‐response theory framework, which includes statistical approaches to link measurement scales or tests and qualitative assessments of the comparability of measures. We will then assess how changes in lifestyle health behaviors impact changes in cognitive outcomes (primary: episodic memory, executive function; secondary: perceptual speed, global cognition). We will examine determinants of health that enhance (or inhibit) these relationships and explore how lifestyle health behaviors may work together. Subgroup analyses will focus on those at greater risk for cognitive decline and/or are historically underrepresented in research.

**Conclusion:**

*InLife* provides a framework for data harmonization efforts with the overall goal of reducing risk of cognitive impairment and ADRD. The *InLife* team continues to welcome data contributions from additional trials to broaden the existing sample. *InLife* has the potential to ultimately inform to clinical guidelines focused on realistic lifestyle health behaviors for cognitive outcomes across diverse aging populations.

